# Predictors of Veterans Health Administration utilization and pain persistence among soldiers treated for postdeployment chronic pain in the Military Health System

**DOI:** 10.1186/s12913-021-06536-8

**Published:** 2021-05-24

**Authors:** Rachel Sayko Adams, Esther L. Meerwijk, Mary Jo Larson, Alex H. S. Harris

**Affiliations:** 1 Heller School for Social Policy & Management, Institute for Behavioral Health, Brandeis University, 415 South Street MS 035, Waltham, MA 02453 USA; 2 Rocky Mountain Mental Illness Research Education and Clinical Center, Veterans Health Administration, 1700 N. Wheeling Street, Aurora, CO 80045 USA; 3 VA Health Services Research & Development, Center for Innovation to Implementation (Ci2i), VA Palo Alto Health Care System, Menlo Park, CA 94025 USA; 4 Department of Surgery, Stanford University, Stanford, CA 94305 USA

**Keywords:** Chronic pain, Veterans, Military, Opioids, Nonpharmacological treatment, Veterans health administration, Postdeployment

## Abstract

**Background:**

Chronic pain presents a significant burden for both federal health care systems designed to serve combat Veterans in the United States (i.e., the Military Health System [MHS] and Veterans Health Administration [VHA]), yet there have been few studies of Veterans with chronic pain that have integrated data from both systems of care. This study examined 1) health care utilization in VHA as an enrollee (i.e., linkage to VHA) after military separation among soldiers with postdeployment chronic pain identified in the MHS, and predictors of linkage, and 2) persistence of chronic pain among those utilizing the VHA.

**Methods:**

Observational, longitudinal study of soldiers returning from a deployment in support of the Afghanistan/Iraq conflicts in fiscal years 2008–2014. The analytic sample included 138,206 active duty soldiers for whom linkage to VHA was determined through FY2019. A Cox proportional hazards model was estimated to examine the effects of demographic characteristics, military history, and MHS clinical characteristics on time to linkage to VHA after separation from the military. Among the subpopulation of soldiers who linked to VHA, we described whether they met criteria for chronic pain in the VHA and pain management treatments received during the first year in VHA.

**Results:**

The majority (79%) of soldiers within the chronic pain cohort linked to VHA after military separation. Significant predictors of VHA linkage included: VHA utilization as a non-enrollee prior to military separation, separating for disability, mental health comorbidities, and being non-Hispanic Black or Hispanic. Soldiers that separated because of misconduct were less likely to link than other soldiers. Soldiers who received nonpharmacological treatments, opioids/tramadol, or mental health treatment in the MHS linked earlier to VHA than soldiers who did not receive these treatments. Among those who enrolled in VHA, during the first year after linking to the VHA, 49.7% of soldiers met criteria for persistent chronic pain in VHA.

**Conclusions:**

The vast majority of soldiers identified with chronic pain in the MHS utilized care within VHA after military separation. Careful coordination of pain management approaches across the MHS and VHA is required to optimize care for soldiers with chronic pain.

**Supplementary Information:**

The online version contains supplementary material available at 10.1186/s12913-021-06536-8.

## Background

Acute and chronic pain are highly prevalent among post-9/11 Veterans who deployed in Operation Enduring Freedom/Operation Iraqi Freedom/Operation New Dawn (OEF/OIF/OND) [1–3]. Complicating pain management and recovery among this population, chronic pain is commonly comorbid with psychological health conditions such as posttraumatic stress disorder (PTSD) or depression, as well as traumatic brain injury [2, 4–6]. In the Military Health System (MHS), the rate of chronic pain diagnoses among military members quadrupled from 2007 to 2014, reaching 68.7 per 10,000 person-years [7], and it is estimated that up to one third of military members will have at least one episode of back pain [8]. Many factors have contributed to the dramatic increase in chronic pain in the MHS, including improved survival rates following combat injury, an increase in repeat deployments, and rigorous physical training demands [9–13]. In the Veterans Health Administration (VHA), acute and chronic pain have been among the most commonly diagnosed medical problems among post-9/11 Veterans [3, 14]. It is clear that chronic pain presents a significant burden for both federal health care systems designed to serve our nation’s combat Veterans. Although efforts have been made to facilitate “seamless transitions” between the MHS and VHA [15], administrative and clinical discontinuities are still a risk for patients moving from one system to the other. Longitudinal examinations of the clinical and treatment trajectories of this high-risk subgroup of post-9/11 Veterans as they transition out of the Department of Defense (DoD) and become eligible to receive services in the VHA could be used to identify gaps in continuity and possible solutions to improve care.

The MHS and VHA have introduced numerous policies and programs to address the burden of chronic pain, with an emphasis on reducing reliance on opioids for pain management due to the risks associated with long-term opioid therapy [14, 16–21]. The Veterans Affairs/Department of Defense clinical practice guidelines recommend using nonpharmacologic treatments (NPT) as first line pain management prior to initiation of opioids [21]. The MHS established specialty pain clinics, implemented a robust patient-centered assessment battery for use in pain specialty care, put forth plans to implement a stepped-care model, and outlined the need to increase referral for NPT [22–24]. The VHA also implemented a stepped-care model for pain management and a national opioid safety initiative to reduce over-reliance on high dose, long-term opioid therapy [16].

While chronic pain and pain management have been studied extensively in both the MHS and VHA since the Afghanistan/Iraq conflicts, there have been few studies of post-9/11 Veterans with chronic pain that have integrated data from both systems of care [25]. Veterans who deployed in OEF/OIF/OND are eligible for free health care at VHA for at least 5 years after separation from the military for reasons other than dishonorable [26]. Despite this entitlement, not all eligible post-9/11 Veterans use VHA health care. A population-based study of active duty soldiers returning from an OEF/OIF/OND deployment in fiscal years 2008–2011 found that only 48% utilized the VHA as an enrollee within 1 year of separation [27]. Those with separation from the military due to disability had the greatest odds of utilizing VHA [27], which may be an indicator for chronic pain for some in this population.

Even though efforts are underway to integrate the electronic medical records between the MHS and VHA, this was not achieved at the time of this study [28]. The vast majority of VHA-utilization studies have little to no information about the health care problems or treatments received while military members were in the MHS. This gap in knowledge limits what we know about post-9/11 Veterans being treated for chronic pain in VHA. Without longitudinal data following military members after they separate from the military and become eligible for VHA service, we do not know the proportion of patients with chronic pain in the MHS that subsequently enrolls in and uses health care in VHA, if pain management treatments received while in the MHS or other characteristics influence the use of VHA, and if chronic pain persists among this population once treated in VHA. To address these gaps in knowledge, this study first describes utilization in VHA, and predictors of utilization, as an enrollee (i.e., linkage to VHA) after separating from the military among a population-based cohort of OEF/OIF/OND active duty soldiers with postdeployment chronic pain identified while in the MHS. Predictors of VHA linkage, all based on MHS data, include: demographics, military history characteristics, clinical characteristics of pain, comorbidities, and pain management treatment (i.e., prescription opioids, receipt of NPT). Second, among the subpopulation of soldiers that linked to VHA, we sought to describe the persistence of chronic pain during the first year in VHA, and pain management receipt in VHA among those with chronic pain that persisted across both systems of care. To our knowledge, this is the first study to follow a post-9/11 Veteran population identified with chronic pain in the MHS as they transitioned into VHA to reveal how persistent chronic pain remains once utilizing care in VHA. These findings have implications for VHA related to planning for workforce capacity and pain management provision, as well as coordination of pain management services between the MHS and VHA.

## Methods

Study data were drawn from the Substance Use and Psychological Injury Combat Study, an observational, longitudinal study of Army soldiers returning from an index deployment in support of the Afghanistan/Iraq conflicts in fiscal years 2008–2014. The SUPIC study integrated data from the MHS and VHA to allow for investigation of postdeployment health, treatment and long-term outcomes across both systems of care [25, 29, 30]. Supported by multiple National Institute of Health grants, to our knowledge, SUPIC remains the only study that prospectively tracks postdeployment soldiers returning from Afghanistan/Iraq deployments as they transition out of the MHS and into VHA.

### Data sources

Deployment data was obtained from the Contingency Tracking System. Demographic characteristics were drawn from the Defense Enrollment Eligibility Records System. Health care diagnoses, utilization and pharmacy data were from the MHS Data Repository inclusive of all outpatient and inpatient claims and encounters from care provided at military treatment facilities (i.e., direct care) as well as care provided by civilian providers and paid for by TRICARE (i.e., purchased care). Pain severity ratings were from the Clinical Data Repository vitals file. Military separation data were provided by the Defense Manpower Data Center. MHS data used in this study were available through September 30, 2015. VHA enrollment (up to and including 2019) and pain diagnoses and pain treatment during the first year after linking were identified in the Corporate Data Warehouse.

*Linkage to VHA* was defined as the first healthcare utilization as an enrollee in VHA, ignoring visits for administrative or immunization purposes only (stopcodes 674 and 710).

### Chronic pain sample

*Chronic pain* was defined as two or more diagnoses at least 90 days apart from the same pain category developed by the project (e.g., back and neck disorders; other musculoskeletal disorders; non-traumatic joint disorders), using International Classification of Disease diagnoses (ICD-9 and ICD-10) as described elsewhere [13]. Two additional non-diagnosis criteria were used to identify chronic pain, including: any 60-day supply of opioids (defined below) prescribed in a 3-month period, or 90-day supply in a 12-month window [25, 31].

From the SUPIC active duty cohort (*n* = 576,516), we included 139,575 soldiers who met criteria for chronic pain that occurred: 1) while in the MHS after returning from the index deployment ending between fiscal years 2008–2014, and 2) prior to separation from the DoD observed till September 30, 2015. The remaining soldiers either did not separate from the military (293,521), did not have any MHS records to determine chronic pain status (17,911), or did not meet criteria for chronic pain (125,509). We excluded soldiers for whom the reason for separation was death (*n* = 748), soldiers with an index deployment duration more than 5 years (*n* = 444), and soldiers who separated during the index deployment (*n* = 241). Accounting for sixty-four soldiers who met more than one of these exclusion criteria, the final analytic sample included 138,206 active duty soldiers for whom linkage to VHA was determined through FY19. Of these, 21,248 (15.4%) soldiers met the opioid day-supply criteria for chronic pain and only 1845 (1.3%) met the chronic pain criteria based on opioid day supply only.

### Exposure measures

#### Pain management treatments

Measures included days supply of opioids and days supply of tramadol (i.e., a weak synthetic opioid), each expressed per year averaged over the length of each soldier’s observation window in the MHS, and receipt of NPT. Prescription opioids included codeine, dihydrocodeine, fentanyl, hydrocodone, hydromorphone, meperidine, methadone, morphine, oxycodone, oxymorphone, and tapentadol – excluding injectable opioids and drugs used to treat addictions (e.g., buprenorphine) [32]. Nonpharmacologic treatments were defined by the study as outpatient services and included acupuncture/dry needling, biofeedback, chiropractic manipulation, massage, exercise therapy, cold laser therapy, spinal manipulation, transcutaneous electrical nerve stimulation and other electrical manipulation, ultrasonography, superficial heat treatment, traction, other physical therapy, and lumbar supports. NPT treatments were identified using Current Procedural Terminology codes and Healthcare Common Procedure Coding System codes [33, 34].

In VHA analysis among soldiers who linked to VHA, we examined receipt of the following pain treatments within the first 365 days after linking to VHA: any prescription opioid and receipt of greater than 30-days supply of opioids (both inclusive of tramadol), receipt of any NPT, and receipt of each individual NPT modality. NPT treatments in VHA were identical to those used in the MHS, with the addition of clinic visits for complementary and integrative health treatment (stop codes 159) or chiropractic care (stop code 436).

### Patient demographic, clinical, and service-related variables

Additional measures derived from MHS data included demographic characteristics (i.e., age, gender, race/ethnicity, marital status), and military history characteristics (i.e., rank, days deployed before and after the index deployment, reason for separation, years of observation in the MHS, and fiscal year of the end of the index deployment). Clinical characteristics included diagnoses for psychological health conditions (i.e., PTSD, adjustment disorders, anxiety disorders [excluding PTSD], alcohol use disorders, and substance use disorders [excluding alcohol]), all based on the Clinical Classifications Software from the Agency for Healthcare Research and Quality, as well as diagnoses for traumatic brain injury defined according to the DoD’s standard traumatic brain injury surveillance case definition [35]. Additional clinical treatments included use of mental health specialty services and use of substance use disorder specialty services based on procedure codes in the MHS, as well as pre-separation non-enrolled VHA utilization.

### Analysis

We completed basic descriptive analyses, including *t*-tests, Mann-Whitney tests, and chi-square tests on demographic characteristics, military history, and MHS clinical history characteristics for those who did and did not link to VHA. A Cox proportional hazards model was estimated to examine the effects of demographic characteristics, military history, and MHS clinical characteristics on time to linkage to VHA after separation from the military. The Cox proportional hazards model does not assume an underlying distribution of time to linkage, but it does assume proportional hazards. We assessed plots for the Schoenfeld residuals against time and found them to support the proportional hazards assumption [36]. Data were right-censored and ties were broken using the Efron approximation, which considers all potential underlying orderings. Because we were particularly interested in prescription opioid use and receipt of NPT among soldiers with chronic pain, we included two-way interaction terms between NPT and other treatments received in the MHS. The Cox proportional hazards interaction model assumes the log hazard functions for different treatment groups to have identical shapes and identical distance at any point in time [36]. We assessed the Kaplan-Meier survival curves for these assumptions and found them to be met. In addition to the Cox proportional hazards model estimates, we present median time to linkage to VHA for soldiers who used substance use disorder or mental health services in the MHS and for soldiers who received pain treatments (7-day and 30-day supply for opioids and tramadol, and receipt of NPT [yes/no]) in the MHS, while controlling other covariates of the model. Among the subpopulation of soldiers who linked to VHA, we described whether they met our criteria for chronic pain in the VHA and whether they received NPT and opioids in the VHA (any opioids [yes/no] and > 30-day supply of opioids) during the first year after linkage.

## Results

Of 138,206 active duty soldiers with chronic pain in the MHS after an index deployment, 109,187 (79%) linked to the VHA after separating from the military. Table 1 shows the demographic characteristics, military history, and MHS clinical characteristics for soldiers who linked and did not link to the VHA.

**Table 1:** Characteristics of soldiers with chronic pain and VHA linkage after separating from the military (*N* = 138,206)

	Linked to VHA		
	No (*n* = 29,019)	Yes (*n* = 109,187)	*P*
**Demographics and Military History**			
Age, median (*IQR*), y	27.0 (22–36)	26.0 (22–35)	< 0.001
Female sex, No. (%)	3475 (12.0)	13,931 (12.8)	< 0.001
Race/ethnicity, No. (%)			< 0.001
White, not Hispanic	18,101 (62.5)	60,961 (55.9)	
Black, not Hispanic	4124 (14.2)	22,144 (20.3)	
Hispanic	2808 (9.7)	11,418 (10.5)	
Other	3935 (13.6)	14,491 (13.3)	
Marital status, No. (%)			< 0.001
Married	18,684 (64.4)	70,900 (64.9)	
Never Married	8531 (29.4)	30,761 (28.2)	
Other	1804 (6.2)	7526 (6.9)	
Index cohort, No. (%)			0.015
2008	6169 (21.3)	22,980 (21.0)	
2009	8756 (30.2)	33,396 (30.6)	
2010	7615 (26.2)	28,504 (26.1)	
2011	3565 (12.3)	13,521 (12.4)	
2012	1932 (6.7)	7392 (6.8)	
2013	712 (2.5)	2600 (2.4)	
2014	270 (0.9)	794 (0.7)	
Rank, No. (%)			< 0.001
Enlisted	24,614 (84.8)	100,982 (92.5)	
Warrant officer	937 (3.2)	2051 (1.9)	
Commissioned Officer	3466 (11.9)	6149 (5.6)	
Days deployed before index, median (*IQR*) ^a^	0.0 (0.0–2.97)	0.0 (0.0–3.32)	< 0.001
Days deployed after index, median (*IQR*) ^a^	0.01 (0.01–1.13)	0.01 (0.01–0.74)	< 0.001
Reason for separating from the military, No. (%)			< 0.001
Expiration of enlistment	7287 (25.1)	24,043 (22.0)	
Disability	5607 (19.3)	40,083 (36.7)	
Retirement	7354 (25.3)	20,237 (18.5)	
Misconduct	1872 (6.5)	4278 (3.9)	
Poor performance	2914 (10.0)	11,352 (10.4)	
Other	3985 (13.7)	9194 (8.4)	
Days after index deployment to separation, mean (*SD*)	1128 (640)	1099 (616)	< 0.001
**Military Health System Clinical History**			
Years of observation in MHS, median (*IQR*)	4.25 (2.50–5.75)	4.50 (2.75–6.00)	< 0.001
Chronic pain categories, No. (%) ^b^			
Peripheral & central nervous system disorders	842 (2.9)	4463 (4.1)	< 0.001
Osteoarthritis	1181 (4.1)	5178 (4.7)	< 0.001
Back & neck disorders	12,082 (41.6)	53,462 (49.0)	< 0.001
Headaches & migraines	2858 (9.8)	17,423 (16.0)	< 0.001
Non-traumatic joint disorders	13,616 (46.9)	54,444 (49.9)	< 0.001
Other musculoskeletal disorders	12,173 (41.9)	47,548 (43.5)	< 0.001
Visceral & pelvic disorders	2211 (7.6)	9717 (8.9)	< 0.001
Wounds & injuries	2261 (7.8)	9371 (8.6)	< 0.001
Acute & post-operative diagnoses, trauma	151 (0.5)	1049 (1.0)	< 0.001
Other diagnoses associated with pain	40 (0.1)	151 (0.1)	1.000
Chronic pain by ICD definition	6778 (23.4)	35,732 (32.7)	< 0.001
Number of chronic pain categories, No. (%)			< 0.001
one	14,126 (48.7)	39,542 (36.2)	
two	7510 (25.9)	28,151 (25.8)	
three	3564 (12.3)	16,669 (15.3)	
four or more	3819 (13.2)	24,825 (22.7)	
Highest reported pain level, No. (%)			< 0.001
None (0)	1090 (3.8)	2394 (2.2)	
Low (1–3)	1794 (6.2)	4105 (3.8)	
Moderate (4–6)	8256 (28.5)	25,420 (23.3)	
Severe (7–10)	17,339 (59.8)	75,366 (69.0)	
Unknown	540 (1.9)	1902 (1.7)	
Mental disorders, No. (%) ^b^			
Adjustment disorder	10,817 (37.3)	55,406 (50.7)	< 0.001
Depressive disorders	7519 (25.9)	42,395 (38.8)	< 0.001
Anxiety disorders	8438 (29.1)	47,131 (43.2)	< 0.001
Posttraumatic stress disorder	5101 (17.6)	34,762 (31.8)	< 0.001
Traumatic brain injury	6626 (22.8)	34,167 (31.3)	< 0.001
Alcohol use disorder	4004 (13.8)	19,896 (18.2)	< 0.001
Substance use disorder	4099 (14.1)	18,813 (17.2)	< 0.001
Used mental health services, No. (%)	19,854 (68.4)	88,108 (80.7)	< 0.001
Used SUD services, No. (%)	1802 (6.2)	8513 (7.8)	< 0.001
Used NPT for pain, No. (%)	22,191 (76.5)	89,344 (81.8)	< 0.001
Number of days with NPT for pain, median (*IQR*) ^c^	2.29 (0.20–6.72)	3.29 (0.67–8.67)	< 0.001
Days supply opioids ^c^, median (*IQR*)	3.70 (0.71–12.7)	6.77 (1.67–24.0)	< 0.001
Days supply tramadol ^c^, median (*IQR*)	0.0 (0.0–3.33)	0.0 (0.0–8.0)	< 0.001

‘Linkage to VHA’ was defined as enrolled and utilized VHA services after separating from the military. Categories are mutually exclusive, unless otherwise indicated. The *P*-value was based on the chi-square test for categorical variables and the nonparametric Mann-Whitney test for all continuous variables, except for “Days after index deployment to separation” which was normally distributed and tested with a *t*-test

*Abbreviations*: *VHA* Veterans Health Administration, *MHS* Military Heath System, *ICD* International Classification of Diseases, *SUD* Substance Use Disorder, *NPT* Nonpharmacological Treatment

^a^ Expressed per 100 days

^b^ Not mutually exclusive

^c^ Expressed per year, averaged over the length of each service member’s observation window in the MHS

The Cox proportional hazards model predicting linkage to VHA in Table 2 included all variables from Table 1 plus interaction terms between NPT and other treatments received in MHS. The model showed statistically significant (*p* <  0.05) contributions of almost all variables in the model. Notably, soldiers who had VHA utilization as a non-enrollee prior to separation, had the highest proportional hazard of linking (HR 2.20), and non-Hispanic Black soldiers and Hispanic soldiers had a higher proportional hazard of linking than non-Hispanic White soldiers (HR 1.27 and 1.14, respectively). Officers had a lower proportional hazard of linking than soldiers who enlisted (HR 0.82 and 0.76 for warrant officers and commissioned officers, respectively). Female soldiers had a lower proportional hazard than males (HR 0.97). Disability as reason of separation had a higher proportional hazard of linking than expiration of enlistment (HR 1.11), similar to separation for poor performance (HR 1.12), whereas retirement, misconduct and other reasons for separating had lower proportional hazards than expiration of enlistment (respectively, HR 0.73, HR 0.80, HR 0.89). Diagnoses associated with wounds and injuries, visceral and pelvic disorders, and other musculoskeletal disorders were not associated with linkage, whereas most other pain conditions had a higher proportional hazard of VA linkage. Except for substance use disorder, all mental health comorbidities had a higher proportional hazard, especially so for PTSD (HR 1.17). Opioids, tramadol, and specialty mental health treatments received in the MHS had higher proportional hazards and a significant interaction with NPT was observed for opioids and tramadol, but the differences were small.

**Table 2:** Cox proportional hazards model predicting VHA linkage among soldiers with chronic pain before military separation (*n* = 138,206) ^a^

	*HR*	95% CI	*P*
Age	1.06	1.05–1.07	< 0.001
Female Sex (Ref. Male)	0.97	0.95–0.99	< 0.01
Race/ethnicity (Ref. non-Hispanic White)			
Black, not Hispanic	1.27	1.25–1.29	< 0.001
Hispanic	1.14	1.12–1.16	< 0.001
Other	1.03	1.01–1.05	< 0.001
Marital Status (Ref. Married)			
Never Married	1.04	1.02–1.05	< 0.001
Other	1.06	1.04–1.09	< 0.001
Index Cohort (Ref. 2008)			
2009	1.03	1.01–1.05	< 0.001
2010	1.07	1.05–1.09	< 0.001
2011	1.10	1.08–1.13	< 0.001
2012	1.14	1.11–1.18	< 0.001
2013	1.23	1.19–1.29	< 0.001
2014	1.12	1.04–1.21	< 0.01
Rank (Ref. Enlisted)			
Warrant Officer	0.82	0.79–0.86	< 0.001
Commissioned Officer	0.76	0.74–0.78	< 0.001
Days deployed before index ^b^	1.01	1.00–1.02	< 0.01
Days deployed after index ^b^	1.02	1.01–1.02	< 0.001
Reason for separating (Ref. Expiration of enlistment)			
Disability	1.11	1.09–1.13	< 0.001
Retirement	0.73	0.71–0.74	< 0.001
Misconduct	0.80	0.78–0.83	< 0.001
Poor Performance	1.12	1.09–1.15	< 0.001
Other	0.89	0.87–0.91	< 0.001
Years of observation in MHS	1.04	1.03–1.04	< 0.001
PNS & CNS disorders	1.05	1.02–1.09	< 0.01
Osteoarthritis	1.04	1.01–1.07	< 0.05
Back & neck disorders	1.04	1.03–1.05	< 0.001
Headaches & migraines	1.09	1.07–1.11	< 0.001
Non-traumatic joint disorders	1.02	1.01–1.03	< 0.01
Other musculoskeletal disorders	1.00	0.99–1.02	0.34
Visceral & pelvic disorders	1.00	0.98–1.02	0.97
Wounds & injuries	0.98	0.96–1.00	0.10
Acute & post-operative diagnoses, trauma	1.09	1.02–1.16	< 0.01
Other diagnoses associated with pain	0.85	0.72–1.00	< 0.05
Chronic pain by ICD definition	1.03	1.02–1.05	< 0.001
Adjustment disorders	1.08	1.06–1.09	< 0.001
Depressive disorders	1.08	1.07–1.10	< 0.001
Anxiety disorders	1.10	1.08–1.11	< 0.001
Post-traumatic stress disorder	1.17	1.16–1.19	< 0.001
Traumatic brain injury	1.05	1.04–1.07	< 0.001
Alcohol use disorder	1.07	1.05–1.09	< 0.001
Substance use disorders	0.97	0.95–0.99	< 0.001
Days supply opioids ^c^	1.08	1.07–1.10	< 0.001
Days supply tramadol ^c^	1.06	1.04–1.07	< 0.001
Used mental health services	1.09	1.05–1.13	< 0.001
Used SUD services	1.05	1.00–1.11	0.06
Receipt of NPT for pain	1.03	0.99–1.06	0.10
Days supply opioids × NPT	0.96	0.95–0.98	< 0.001
Days supply tramadol × NPT	0.98	0.97–1.00	< 0.05
Used mental health services × NPT	1.04	1.00–1.08	0.05
Used SUD services × NPT	0.92	0.87–0.98	< 0.01
Pre-separation non-enrolled VHA utilization	2.20	2.17–2.23	< 0.001

‘Linkage to VHA’ was defined as enrolled and utilized VHA services after separating from the military

*Abbreviations*: *VHA* Veterans Health Administration, *HR* Hazard Ratio, *CI* Confidence Interval, *MHS* Military Heath System, *PNS* Peripheral Nervous System, *CNS* Central Nervous System, *ICD* International Classification of Diseases, *SUD* Substance Use Disorder, *NPT* Nonpharmacological Treatment

^a^ If no unit or reference category is indicated, variable is dichotomous [No/Yes] with ‘No’ as reference

^b^ Expressed per 100 days

^c^ Expressed per year, averaged over the length of each service member’s observation window in the MHS

**Fig. 1:**
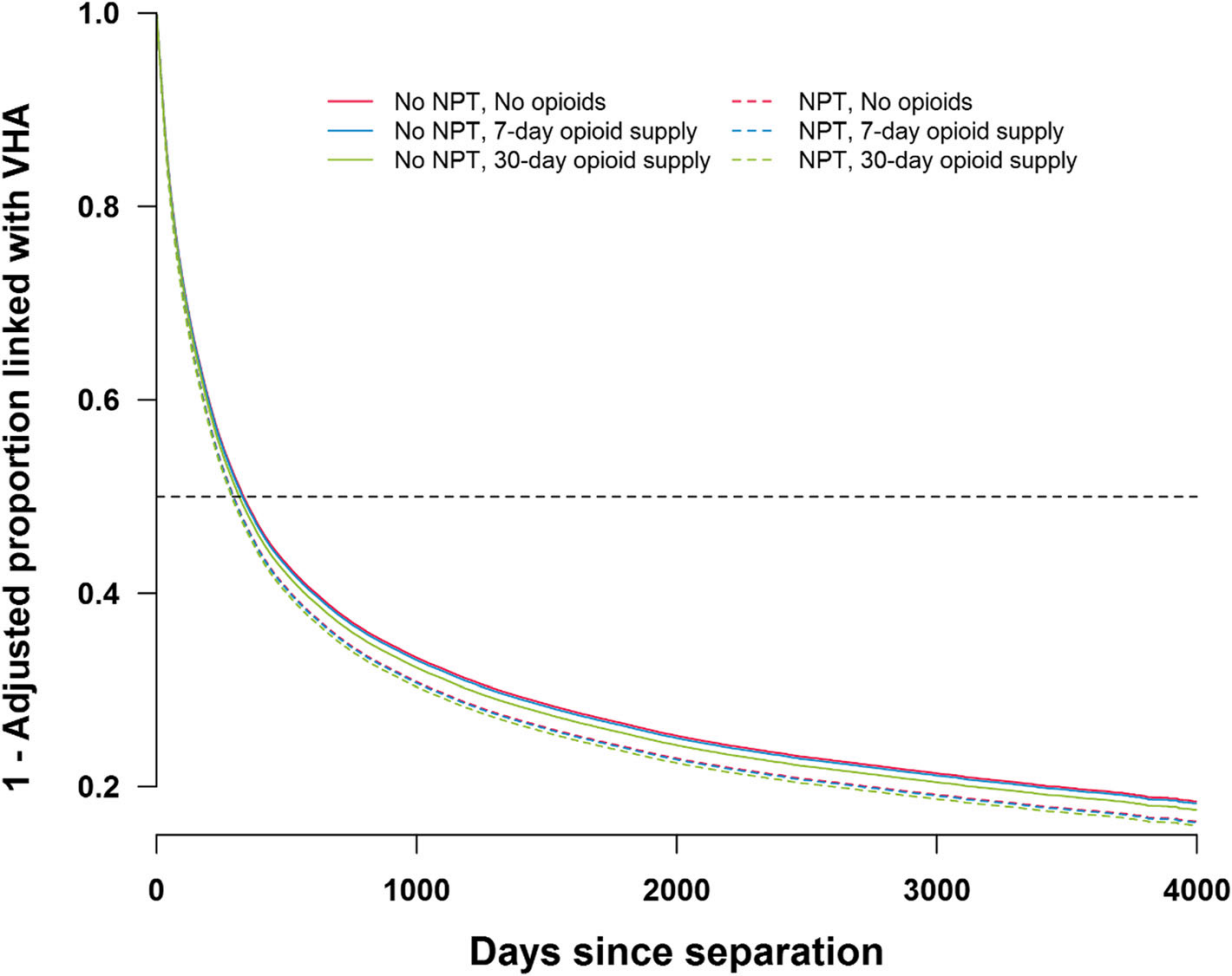
Adjusted linkage to VHA for U.S. active duty soldiers with chronic pain by receipt of nonpharmacological treatments (NPT) and opioid day supply in the MHS. ‘Linkage to VHA’ was defined as enrolled and utilized VHA services after separating from the military. Curves apply to male non-Hispanic white soldiers with chronic pain who were married, who enlisted, whose index deployment ended in FY08 and who separated from the military for reason of expiration of enlistment. Continuous covariates in the model were set to their median value and other covariates were set to their mean to reflect proportions. The ‘No opioids’ line is obscured by the ‘7-day opioid supply’ line for those who did receive NPT (dashed lines)

By way of illustration, Fig. 1 shows the (small) adjusted effect of receiving NPT for increasing average annual days supply of opioids (0, 7, and 30 day supply), such that soldiers who received NPT were more likely to link than soldiers who did not receive NPT at corresponding levels of opioid supply and while keeping other covariates constant.

Not included in the Cox proportional hazards model, to avoid collinearity with the pain conditions themselves, were the number of pain conditions present for each soldier (see Table 1). The proportion of soldiers who met criteria for more than four conditions associated with pain was significantly higher among those who linked to VHA compared to those who did not link (22.7% vs. 13.2%, *p* < .001), whereas the proportion of soldiers who met criteria for only one condition was significantly lower among those who linked to VHA (36.2% vs. 48.7%, *p* < .001).

The overall adjusted median time between separation and linkage was 301 days, however time-to-linkage was associated with MHS treatment history. Table 3 shows the adjusted median time to linkage for male non-Hispanic white soldiers with chronic pain who were married, enlisted, whose index deployment ended in FY08, and who separated from the military for reason of expiration of enlistment. Continuous covariates were set to their median value and other covariates were set to their mean to reflect proportions. Overall, soldiers who received NPT, opioids/tramadol, or services for mental health issues in the MHS linked earlier to VHA than soldiers who did not receive treatments (see Table 3), and the effect was more pronounced for receipt of NPT (median time to linkage 36 days lower for those who received NPT) and use of mental health specialty services (median time to linkage 67 days lower for those who used mental health services).

**Table 3:** Adjusted time between military separation and VHA linkage among soldiers with chronic pain by treatment ^a^

Treatment in MHS	Median time-to-linkage in days (95% CI)
Day supply opioids ^b^	
None	303 (292–315)
7-day supply	301 (290–312)
30-day supply	294 (284–305)
Day supply tramadol ^b^	
None	301 (290–313)
7-day supply	297 (286–308)
30-day supply	282 (272–293)
Receipt of NPT	
No	331 (317–347)
Yes	295 (284–306)
Services for mental health issues	
No	355 (339–373)
Yes	288 (278–299)
Services for substance use issues	
No	301 (290–312)
Yes	308 (292–326)

‘Linkage to VHA’ was defined as enrolled and utilized VHA services after separating from the military. Events show the number of soldiers who linked

*Abbreviations*: *VHA* Veterans Health Administration, *MHS* Military Health System, *NPT* Nonpharmacological Treatment, *CI* Confidence Interval

^a^ Time to linkage is adjusted for covariates in the Cox proportional hazards model. Data shown are for male non-Hispanic white soldiers with chronic pain who were married, who enlisted, whose index deployment ended in FY08 and who separated from the military for reason of expiration of enlistment. Continuous covariates in the model were set to their median value and other covariates were set to their mean to reflect proportions

^b^ Expressed per year

During the first year after linking to the VHA, 49.7% of soldiers (*n* = 54,309) met criteria for persistent chronic pain in VHA while the remainder appeared to have pain resolved (see Table 4 and Additional file 1). The most common types of chronic pain in VHA were back and neck disorders, non-traumatic joint disorders, other musculoskeletal disorders, and headaches and migraines. Among soldiers who linked and met criteria for persistent chronic pain in the VHA, 41.0% received one or more opioid prescriptions and 37.4% received an opioid supply of more than 30-days during the first year after linking. Subgroups treated for certain types of chronic pain were more likely to receive more than 30-days supply of opioids (i.e., other diagnoses associated with pain; wounds and injuries; chronic pain by ICD definition; visceral and pelvic disorders; and peripheral and central nervous system disorders). Over one-third (38.1%) of soldiers with persistent chronic pain received at least one NPT treatment in the first year after linking to VHA, with the most common NPT treatments being exercise therapy (26.9%), other physical therapy (19.5%), transcutaneous electrical nerve stimulation /electrical modulation (7.1%), massage (7.0%), and superficial heat treatment (6.8%).

**Table 4:** Treatments received in VHA during the 365-days after linkage among soldiers with persistent chronic pain^a^ (*n* = 54,309)

	Any Chronic Pain	Back & neck disorders	Headaches & migraines	Non-traumatic joint disorders	Other musculoskeletal disorders	Chronic pain by ICD definition ^b^
	(*n* = 54,309)	(*n* = 31,732)	(*n* = 14,053)	(*n* = 18,854)	(*n* = 17,034)	(*n* = 6016)
Nonpharmacological treatments						
Any NPT	20,670 (38.1)	14,106 (44.5)	5901 (42.0)	8653 (45.7)	8445 (49.6)	2848 (47.3)
Exercise therapy	14,607 (26.9)	9706 (30.6)	4251 (30.2)	6463 (34.1)	6079 (35.7)	2111 (35.1)
Other physical therapy	10,571 (19.5)	7299 (23.0)	2981 (21.2)	4988 (26.3)	4682 (27.5)	1365 (22.7)
Chiropractic care	2005 (3.7)	1805 (5.7)	595 (4.2)	631 (3.3)	1001 (5.9)	307 (5.1)
TENS/electrical modulation	3848 (7.1)	3049 (9.6)	1282 (9.1)	1677 (8.8)	1769 (10.4)	552 (9.2)
Massage	3796 (7.0)	2808 (8.9)	1211 (8.6)	1688 (8.9)	1992 (11.7)	521 (8.7)
Superficial heat treatment	3672 (6.8)	2661 (8.4)	1157 (8.2)	1798 (9.5)	1960 (11.5)	479 (8.0)
Opioids						
Any opioid prescription	22,253 (41.0)	13,364 (42.1)	5084 (36.2)	7916 (41.8)	7423 (43.6)	3008 (50.0)
> 30-day supply	20,291 (37.4)	12,175 (38.4)	4641 (33.0)	7168 (37.8)	6760 (39.7)	2812 (46.7)

Notes: ‘Linkage to VHA’ was defined as enrolled and utilized VHA services after separating from the military. Chronic pain was determined based on the soldiers’ first year of utilization after linking to VHA and categories are not mutually exclusive. The sample includes soldiers who met criteria for chronic pain during the first 365 days in the VHA (i.e., persistent chronic pain)

*Abbreviations*: *VHA* Veterans Health Administration, *ICD* International Classification of Diseases, *NPT* Nonpharmacological Treatment, *TENS* Transcutaneous Electrical Nerve Stimulation

^a^ Chronic pain categories not as common, and nonpharmacological treatment modalities with less than 4% utilization, are shown in Additional File 1

^b^ Includes ICD-9 diagnosis codes 338.2 (chronic pain) and 338.4 (chronic pain syndrome) and ICD-10 diagnosis codes G89.2 (chronic pain) and G89.4 (chronic pain syndrome)

## Discussion

We found that among a population-based sample of 138,206 active duty soldiers with chronic pain identified in the MHS after return from deployment in fiscal years 2008–2014 who separated from military duty, 79% utilized VHA health care as an enrollee. This finding was striking and demonstrates that the vast majority of post-9/11 Veterans with chronic pain identified in the MHS rely, at least in part, utilize VHA for health care after separating from the military. By comparison, a prior study that examined linkage to VHA in the first year after military separation among an Army population of active duty soldiers not restricted to a chronic pain cohort [27], found lower linkage to VHA (48%). Our present study had a longer observation window; however, the difference in observation time does not appear to explain the dramatically higher proportion of linkage to VHA. More research is needed to understand if this higher frequency of linkage to VHA among post-9/11 Veterans with postdeployment chronic pain reflects greater interest and medical need in connecting to VHA services, is related to better coordination of care, or occurs for another reason.

Our model examining the factors associated with linking to VHA revealed important information particularly relevant for the 21% of the sample that did not enroll in VHA. We found that Black/non-Hispanic and Hispanic soldiers were more likely to use VHA health care as an enrollee than White/non-Hispanic soldiers, and officers were less likely to link to VHA compared to enlisted soldiers, similar to prior analyses with an Army active duty population not restricted to those with chronic pain [27]. These differences may reflect differences in employment opportunities after leaving the military and access to employer-provided health care. Female soldiers were less likely to link to VHA compared to males, a finding that differed from prior research that examined the broader active duty cohort not restricted to a chronic pain sample which found that female active duty soldiers were more likely to link to VHA than males [27]. This new finding is consistent with research that finds access to women-specific services is not consistently available across all VHA facilities [37], and may reflect differences in the type of pain condition among males and females [3]. Additional research on gender and race/ethnicity differences among MHS patients with chronic pain who do not link to the VHA is warranted.

The most important predictor for linkage to VHA was pre-separation utilization of VHA as a non-enrollee, defined as VHA hospitalization or outpatient visits, excluding visits for administration purposes or vaccinations only. VHA health care is available to active duty military members by referral from military treatment facilities or upon emergency situations through Sharing Arrangements between DoD and VHA, or through TRICARE coverage [38]. This finding may imply that active duty soldiers who used VHA health care while still in military service had a positive experience and were more inclined to enrollee in VHA post-separation. It also may reflect soldiers who had more severe or complicated health care needs while in the military and required additional services provided by VHA (e.g., VHA polytrauma system of care) [4], providing more support for the theory that Veterans who are sicker and have more complicated health care needs seek out VHA health care.

Reason for separation was among the most important predictors for linking to VHA. Compared to soldiers who separated from the military due to expiration of enlistment, soldiers separating due to disability or poor performance had a higher likelihood of linking to VHA, while those who separated due to misconduct or retirement were less likely to link to VHA. Soldiers who retire from the military continue to receive health care benefits funded by the DoD (i.e., TRICARE health plan). Our prior analyses not restricted to a chronic pain cohort also found that separation due to disability was among the strongest predictors of VHA linkage among active duty soldiers [27]. It is encouraging that VHA is attracting the population with military-related disabilities, given this population may have specialized needs and comorbidities not as easily addressed by civilian providers. The finding that soldiers who separated due to poor performance were more likely to link to VHA may be capturing soldiers who had job impairments due to their chronic pain, and/or may demonstrate a greater preference or need among this group to use VHA health care. It is well documented that a critical barrier to VHA care is receipt of an other-than-honorable discharge [39], a discharge that prohibits utilization of VHA care (with some exceptions). While our study did not have access to data that characterized separations as honorable or other-than-honorable, most likely other-than-honorable discharges are among those separating for misconduct and other discharges, groups where we found lower likelihood of linkage to VHA. A Government Accountability Office [39] report found that 62% of military members who separated from the military between 2011 and 2015 had been diagnosed with PTSD, traumatic brain injury, or other psychological health conditions (e.g., adjustment disorder, alcohol use disorder) in the 2 years before separation; and 23% of this group received an other-than-honorable characterization of services making them potentially ineligible for VHA services. In our study, only substance use disorder diagnoses were associated with reduced likelihood of linking to VHA, perhaps partially explained because use of illicit drugs is considered misconduct in the military and may have military career-ending ramifications [40]. Our results suggest that the group of military members with chronic pain who were separated from service for misconduct face diminished access to VHA health care for military-related conditions.

We found that certain chronic pain diagnoses received in the MHS were associated with increased likelihood of linking to VHA, with the greatest likelihood of linking among soldiers with the following conditions: headaches and migraines, acute and post-operative diagnoses or trauma, chronic pain by ICD definition, and peripheral and central nervous system disorders. Pain management and other treatments received while in the MHS influenced both likelihood of linking to VHA and time to linkage. Overall, receipt of NPT, pharmacologic treatments (i.e., opioids, tramadol), mental health services and substance use disorder services each individually increased the likelihood of linkage to VHA, compared to soldiers who did not receive treatments or who received a lower dose of treatment. We found that soldiers with chronic pain who utilized both opioid medications and NPT for pain management while in the MHS were more likely to link to VHA and to link sooner after separating. One possible explanation is that this group has more unresolved pain that requires ongoing healthcare; soldiers receiving prescription opioids in particular may be motivated to link to the VHA so that they can continue with their treatment. It is also plausible that use of both NPT and opioid medications is a signal of more complex pain. In our previous work we found positive benefits to receiving NPT while in the MHS; NPT was associated with reduced risk for numerous adverse long-term outcomes in the VHA (e.g., alcohol and/or drug use disorders, accidental drug poisoning, suicidal ideation, and self-inflicted injuries including suicide attempt) [25]. In a prior study of soldiers treated for back pain episodes in the MHS, we found that NPT utilization within 30 days of back pain diagnosis was associated with reduced reliance on opioid prescribing, reduced duty limitations, and reduced pain-related hospitalization and emergency department use during follow-up, while early opioid utilization was associated with more negative outcomes [34].

Approximately half of sample soldiers (49.7%, *n* = 54,309) met the definition for chronic pain within the first 12 months in VHA. So, the burden of military-acquired chronic pain can persist for several years after deployment, demonstrating the importance of easing the transition from MHS into VHA and focusing on strategies that will promote continuity of care. Among this cohort, more than one-third were prescribed opioids and received more than 30-days supply in the first 12 months in VHA. This finding raises concern about the potential for long-term opioid use and concern about reliance on opioids within the VHA as a pain strategy. As days-supply of opioids increases, so does risk for progression to long-term opioid therapy and associated risks for development of dependence and opioid use disorder [20]. Thus, it is critical that the VHA anticipate the NPT needs of new enrollees with chronic pain. This anticipation implies early medical screening of new VHA enrollees for a history of chronic pain, and medical inquiry about prior pain management strategies including NPT. Clinical guidelines, the VHA stepped model of care, and opioid safety initiatives at VHA each suggest that patients start on NPT and that opioids are ineffective in long-term management of chronic pain [16, 21]. The high likelihood of opioid prescribing for more than 30 days also implies that VHA providers may need more training and support around opioid tapering [41]. Patients who have received opioid medications while in the MHS may request continuation of this treatment, despite evidence that indicates it will be ineffective in the long-run [20].

Among soldiers who linked and met criteria for persistent chronic pain in the VHA, over one-third (38.1%) received at least one NPT treatment in the first year after linking to VHA, with the most common NPT treatments being exercise therapy (26.9%) and other physical therapy (19.5%). Most NPT modalities were infrequently used. While it is plausible that VHA enrollees may be receiving more NPT than recorded in the electronic medical record [42], this finding is concerning. It implies that the likelihood of receiving NPT for chronic pain is about the same as the likelihood of receiving an opioid for 30 days or longer. Future research should focus on the barriers to early receipt of NPT among new enrollees to the VHA.

While there are unique features in the United States related to the organization, delivery and financing of health care in the MHS and VHA, many other countries around the world have military medical systems [43] and need to support the transition of military members into other health care delivery systems once ending military service. Our findings suggest that future study may be warranted in other countries related to transition of care and pain management for military members with chronic pain as they leave the military medical system and transition back into other medical settings.

## Limitations

Although our focus was on patients with chronic pain, this group commonly had complex comorbidities. Because of our study design (lack of comparator, and observational approach), we cannot determine if pain, other comorbidities, or previous treatment experiences might explain the higher VHA linkage found in this study compared to those in our previous studies. These observational analyses identified that certain groups of people were less likely to link to VHA, most notably soldiers who separated for reasons of misconduct, but cannot determine the underlying causes. Furthermore, our data only included NPT delivered by MHS and VHA providers. Some soldiers may have utilized NPT in other community-settings or own their own.

## Conclusions

This study presents unique analysis that combined MHS and VHA health care data on a population of active duty soldiers who had separated from the military. We found that the vast majority of soldiers identified with chronic pain in the MHS had enrolled in and utilized care within VHA. This linkage occurred more frequently and in fewer months among soldiers who had received opioid prescriptions and NPT services while in the MHS. Thus, to optimize care for these soldiers with chronic pain, requires careful coordination of pain management approaches across the MHS and VHA. Further, we identified that soldiers with chronic pain who separated because of misconduct were much less likely to link to the VHA; and female soldiers and those a substance use diagnosis use diagnosis were slightly less likely to link than other solders. Programmatic changes may be required to accommodate the needs of underserved females and soldiers with substance use disorders. The policies that prohibit VHA enrollment of military members with less than honorable discharge appear to have an unintended consequence of restricting access to pain management services for some military members with postdeployment chronic pain.

## Supplementary Information

**Additional file 1. **VHA treatments received during the first 365 days after linking among U.S. Army soldiers who met chronic pain criteria in VHA (*n* = 54,309). Chronic pain was determined based on the soldiers’ first year of utilization after linking to VHA and categories are not mutually exclusive. Pain treatments include nonpharmacological treatments and opioids.

## Data Availability

The Defense Health Agency’s Privacy and Civil Liberties Office provided access to Department of Defense (DoD) data. The datasets generated and analyzed during the current study are not publicly available according to our Data Sharing Agreement, and are governed by the Defense Health Agency and Veterans Health Affairs.

## Abbreviations

DoD: Department of Defense
MHS: Military Health System
OEF/OIF/OND: Operation Enduring Freedom/Operation Iraqi Freedom/Operation New Dawn
NPT: Nonpharmacological treatments
PTSD: Posttraumatic stress disorder
VHA: Veterans Health Administration
