# Supplementary material for: Predictors of Veterans Health Administration utilization and pain persistence among soldiers treated for postdeployment chronic pain in the Military Health System
Source: BMC Health Serv Res. 2021 May 24;21:494. doi: 10.1186/s12913-021-06536-8 (PMC8145830; doi:10.1186/s12913-021-06536-8)
Supplement: Supplementary file 1 — Additional file 1. VHA treatments received during the first 365 days after linking among U.S. Army soldiers who met chronic pain criteria in VHA (n = 54,309). Chronic pain was determined based on the soldiers’ first year of utilization after linking to VHA and categories are not mutually exclusive. Pain treatments include nonpharmacological treatments and opioids. [file 12913_2021_6536_MOESM1_ESM.docx]

Additional File 1. VHA treatments received during the first 365 days after linking among U.S. Army soldiers who met chronic pain criteria in VHA (*n* = 54309)

|  | Any chronic Pain | Peripheral & CNS disorders | Osteoarthritis | Back & neck disorders | Headaches & migraines | Non-traumatic joint disorders | Other musculoskeletal disorders | Visceral & pelvic disorders | Wounds & injuries | Acute & post-operative diagnoses, trauma | Other diagnoses associated with pain | Chronic pain by ICD definition ^a^ |
| --- | --- | --- | --- | --- | --- | --- | --- | --- | --- | --- | --- | --- |
|  | (n=54309) | (n=1720) | (n=2750) | (n=31732) | (n=14053) | (n=18854) | (n=17034) | (n=2023) | (n=1412) | (n=29) | (n=551) | (n=6016) |
| Nonpharmacological treatments  Any NPT  Exercise therapy  Other physical therapy  Chiropractic care  TENS/electrical modulation  Massage  Spinal manipulation  Acupuncture or dry needling  Biofeedback  Superficial heat treatment  Ultrasonography  Lumbar supports  Traction  Cold laser therapy  CIH clinic visits | 20670 (38.1)  14607 (26.9)  10571 (19.5)  2005 (3.7)  3848 (7.1)  3796 (7.0)  133 (0.2)  791 (1.5)  405 (0.7)  3672 (6.8)  1713 (3.2)  462 (0.9)  1210 (2.2)  317 (0.6)  760 (1.4) | 838 (48.7)  609 (35.4)  489 (28.4)  72 (4.2)  182 (10.6)  183 (10.6)  6 (0.3)  37 (2.2)  10 (0.6)  178 (10.3)  95 (5.5)  17 (1.0)  68 (4.0)  22 (1.3)  43 (2.5) | 1128 (41.0)  834 (30.3)  625 (22.7)  72 (2.6)  226 (8.2)  187 (6.8)  6 (0.2)  29 (1.1)  26 (0.9)  239 (8.7)  105 (3.8)  30 (1.1)  63 (2.3)  18 (0.7)  35 (1.3) | 14,106 (44.5)  9706 (30.6)  7299 (23.0)  1805 (5.7)  3049 (9.6)  2808 (8.9)  110 (0.3)  668 (2.1)  263 (0.8)  2661 (8.4)  1139 (3.6)  422 (1.3)  1104 (3.5)  198 (0.6)  602 (1.9) | 5901 (42.0)  4251 (30.2)  2981 (21.2)  595 (4.2)  1282 (9.1)  1211 (8.6)  37 (0.3)  358 (2.5)  186 (1.3)  1157 (8.2)  515 (3.7)  135 (1.0)  413 (2.9)  92 (0.7)  320 (2.3) | 8653 (45.7)  6463 (34.1)  4988 (26.3)  631 (3.3)  1677 (8.8)  1688 (8.9)  44 (0.2)  303 (1.6)  176 (0.9)  1798 (9.5)  936 (4.9)  160 (0.8)  395 (2.1)  164 (0.9)  282 (1.5) | 8445 (49.6)  6079 (35.7)  4682 (27.5)  1001 (5.9)  1769 (10.4)  1992 (11.7)  53 (0.3)  418 (2.5)  173 (1.0)  1960 (11.5)  957 (5.6)  149 (0.9)  619 (3.6)  154 (0.9)  361 (2.1) | 767 (37.9)  567 (28.0)  399 (19.7)  66 (3.3)  146 (7.2)  143 (7.1)  0 (0.0)  38 (1.9)  15 (0.7)  123 (6.1)  71 (3.5)  18 (0.9)  45 (2.2)  12 (0.6)  35 (1.7) | 737 (52.2)  581 (41.2)  366 (25.9)  56 (4.0)  158 (11.2)  156 (11.0)  2 (0.1)  21 (1.5)  18 (1.3)  148 (10.5)  83 (5.9)  12 (0.8)  31 (2.2)  26 (1.8)  26 (1.8) | 13 (44.8)  12 (41.4)  1 (3.4)  2 (6.9)  2 (6.9)  4 (13.8)  0 (0.0)  2 (6.9)  0 (0.0)  4 (13.8)  1 (3.4)  0 (0.0)  0 (0.0)  0 (0.0)  1 (3.4) | 288 (52.3)  218 (39.6)  170 (30.9)  37 (6.7)  62 (11.3)  69 (12.5)  3 (0.5)  36 (6.5)  11 (2.0)  58 (10.5)  27 (4.9)  7 (1.3)  17 (3.1)  5 (0.9)  24 (4.4) | 2848 (47.3)  2111 (35.1)  1365 (22.7)  307 (5.1)  552 (9.2)  521 (8.7)  20 (0.3)  191 (3.2)  73 (1.2)  479 (8.0)  230 (3.8)  74 (1.2)  162 (2.7)  40 (0.7)  198 (3.3) |
| Opioids  any opioid prescription  > 30-day supply | 22253 (41.0)  20291 (37.4) | 789 (45.9)  718 (41.7) | 1096 (39.9)  1013 (36.8) | 13,364 (42.1)  12,175 (38.4) | 5084 (36.2)  4641 (33.0) | 7916 (41.8)  7168 (37.8) | 7423 (43.6)  6760 (39.7) | 1032 (51.0)  917 (45.3) | 746 (52.8)  691 (48.9) | 15 (51.7)  15 (51.7) | 312 (56.6)  310 (56.3) | 3008 (50.0)  2812 (46.7) |

‘Linkage to VHA’ was defined as enrolled and utilized VHA services after separating from the military. Chronic pain was determined based on the soldiers’ first year of utilization after linking to VHA and categories are not mutually exclusive.

^a^ Includes ICD-9 diagnosis codes 338.2 (chronic pain) and 338.4 (chronic pain syndrome) and ICD-10 diagnosis codes G89.2 (chronic pain) and G89.4 (chronic pain syndrome).

Abbreviations: VHA: Veterans Health Administration; CNS: Central Nervous System; ICD: International Classification of Diseases; NPT: Nonpharmacological Treatment; TENS: Transcutaneous Electrical Nerve Stimulation; CIH: Complementary & Integrative Health.
